# A Medico-Religious Charity—The Guild of Mercy

**Published:** 1900-03

**Authors:** W. Thornton Parker

**Affiliations:** Westboro, Mass.


### A MEDICO-RELIGIOUS CHARITY — THE GUILD OF MERCY.

BY W. THORNTON PARKER, M.D.,

WESTBORO, MASS.

We read so much of late concerning the abuse of medical charity, that it must be relief for those who wish to do good professionally to learn that a somewhat new avenue has been opened in the work of the Guild of Mercy.

There can be no question as to legitimacy of the ministrations for the poor and suffering which the members of this society have so unselfishly undertaken.

Who has not felt, when visiting the unfortunate and the ignorant, the yearning for that knowledge which, while ministering to diseased and enfeebled bodies, would enable one to encourage and uplift perishing souls? We must often recall the power of that loving one whose hand so often released the suffering body from pain, before uttering the soul-healing command, "Go, and sin no more."

The good physician often improves his many opportunities to encourage the spiritual care of these patients. He can discourage vice and evil speaking; he can call to mind the greatest act of physical suffering ever endured on this earth, and he can to those sinking under sadness tell of the broken heart of the Son of Man on Calvary. Our larger cities in a great measure supply medical charities through hospitals and dispensaries, and much of that work is perhaps overdone, to the extent of creating an abuse, but as a rule it is not to the cities that our home missionaries are sent, and a very meager acquaintance with outlying fields will convince one of the immense advantage such adjuncts offer to spiritual agencies. Not only are physicians in such places generally less accessible, but how often does poverty close the door of hut or cabin to medical aid, as to many other blessings, when they are desperately needed. What might not be effected through a medical missionary in such communities as a fellow-laborer to ministers of the gospel, or combining the two functions in one? How often in fact might not deadened souls be awakened to a higher conception of the spiritual life were the message of God's love and law accompanied by instruction and aid securing an improved physical life. For, true as it undoubtedly is, that sin is the father of disease, it is none the less true, that in its turn disease is the promoter of sin. The languid frame, the dejected mind, and the weakened will are but poor weapons with which to meet man's invisible foes. Is there not a call, therefore, for ministers of the body as well as of the soul? And how shall they minister except they be sent.

The medical deacon or missionary must first of all have the divine call. He must be ready to declare that he truly believes that God has called him to give himself body and soul and all that he is to the Church, that he may succor and comfort Christ's poor sick. The medical profession ought to be able at the present day —overcrowded as it is—to supply excellent material for this work. Men are needed who have thorough professional training; especially hospital experience is desirable; military medical service is a good school for the medical deacon. Discipline, habits of obedience, care in the preparation of reports, economy in the administration of medicines, surgical appliances, and general disbursements. Good health, patience, fortitude and a deep religious faith—all these are requisites in this important work. It has been proposed by a denomination of Christians zealous in medical missionary work, to educate young people as medical missionaries, combining theological with a quasi-medical training! *This seems to be a mistake.* It is not true that an indifferent physician will make a good enough medical missionary! The Church has a right to demand the highest talents, and such are, or should be, always ready to be placed at her feet. A man who has practiced medicine for many years, after a thorough medical training and *then* receives the *call,* is, all other things being equal, better suited for this work than one who seeks the place before graduation, or, worse still, before beginning the study of medicine. The field for the medical deacon is a very large one; our cities, towns and mission fields could absorb largely all the men we could send. The need is constantly increasing and the work demanding laborers. The Church is busy providing for the clergy. Medical charities must be developed among laymen.

For the past ten years there has existed a medico-religious society now known as the Guild of Mercy, which has the following objects:

1. To minister to the sorrowing, the suffering, the sick and the dying.

2. To visit prisoners and those under restraint, and to aid those unjustly accused and condemned.

3. To encourage medical missions, domestic and foreign.

Through the aid of medical missions invaluable opportunities are afforded for the extension of Christ's Kingdom. More so than any other, the profession of medicine, so highly favored by divine recognition, should stand ready to aid the Church not only by affording scientific witness to the truth as it is in Jesus, but by practical works of mercy for his sake—continually remembering the example of him who went about doing good. Recalling the life of our merciful Saviour while on earth, we cannot fail to see how he blessed the art of healing and how frequently he made use of curing diseased bodies in order to reach and save souls. There are few households among our people to which each year does not bring especial cause for thanksgiving, either for the blessing of uninterrupted health, or else health restored through medical aid under God's blessing. Should not these remember the shadowed homes, the suffering ones cut off in a large measure from such aid, and to whom a few words of skill and kindly counsel might bring life and sunshine? Who has not felt, when released through the ministry of the healing art from a bed of suffering, the yearning to send to other bedsides the same inestimable benefit? It is one of the objects of the Guild of Mercy to provide an opportunity for such impulses of the heart.

This fraternity of mercy is designed to promote the spirit that is suggested by the name.

It is impossible to put into the form of absolute rules the method of work aimed at; at the same time it can be made an obligation on the part of members to be on the lookout for occasions to exercise the virtue of mercy to all who are in suffering or affliction. A beloved Bishop of the Episcopal Church, writing of this Guild ten years ago, used these words: "It is a noble work, giving emphasis to the life of all medical men who are so well known for charity everywhere. It embodies in a very peculiar sense the true spirit of the gospel of Jesus. Would that all our medical men might be physicians of souls as well as healers of bodily malady. If I had the means at my command I would contribute largely to the support of this good work and wish it full success and the benediction of the Saviour."

Members of the Guild of Mercy who are physicians and nurses are expected to offer their services to the clergymen of the parish in which they reside to look after special cases and to be ready at all times to give their services as the Church may have need of them. Briefly stated, the Guild of Mercy is a society of medical practitioners, students, nurses and others interested in the work of mercy for the sorrowing and suffering.

The Guild comprises an order of brothers and associates, communicants of the Anglo-Catholic Church (known in this country as Episcopalians).

The emblem of the order is the crucifix, on the reverse arms of which appears the word, "*Misericordia.*"

The death's head and words "*Memento Mori*" are also used as emblems.

*Members are pledged to faithfully strive to carry out the objects of the Guild with "charity, humility and fortitude"; also to preserve in secrecy all the works of mercy they may be able to perform, and also not to disclose the names of the members of the Guild.*

*All who desire, for any cause, to devote the remainder of their lives to deeds of charity—all—rich or poor, saint or sinner, are welcome to membership under the restrictions already mentioned. Those whose hearts have been touched by sorrow or misfortune, or who, through charitableness, seek to offer help to sufferers, will find in this organization a help to such a noble aim. The work is crippled because the Guild is poor. Money is needed to print the Breviary of the Guild of Mercy, a book of devotion and a manual for members. At least one hundred and fifty dollars is needed for this alone.*

The members are scattered from the Atlantic to the Pacific, in the South seas and in far-away Korea, but it is hoped that a new life of usefulness is in future, and those who are qualified and in earnest are sure of a welcome.

*February 14, 1900.*

